# Molecular analysis of *CHX10* and *MFRP* in Chinese subjects with primary angle closure glaucoma and short axial length eyes

**Published:** 2008-07-17

**Authors:** Tin Aung, Marcus C.C. Lim, Tina T.L. Wong, Anbupalam Thalamuthu, Victor H.K. Yong, Divya Venkataraman, Anandalakshmi Venkatraman, Paul T.K. Chew, Eranga N. Vithana

**Affiliations:** 1 Singapore National Eye Centre, Singapore; 2 Singapore Eye Research Institute, Singapore; 3 Yong Loo Lin School of Medicine, National University of Singapore, Singapore; 4 Genome Institute of Singapore, Singapore; 5 National University Hospital, Singapore

## Abstract

**Purpose:**

The genetic basis of primary angle closure glaucoma (PACG) has yet to be elucidated. Ocular characteristics related to PACG such as short hyperopic eyes with shallow anterior chambers suggest the involvement of genes that regulate ocular size. *CHX10*, a retinal homeobox gene associated with microphthalmia, and *MFRP*, the membrane-type frizzled-related protein gene underlying recessive nanophthalmos, represent good candidate genes for PACG due to the association with small eyes. To investigate the possible involvement of *CHX10* and *MFRP* in PACG, we sequenced both genes in PACG patients with small ocular dimensions.

**Methods:**

One hundred and eight Chinese patients with axial lengths measuring 22.50 mm or less were selected for analysis. Ninety-three age- and ethnically-matched control subjects were also screened. Genomic DNA was extracted from leukocytes of peripheral blood samples, and 	the exons of *CHX10* and *MFRP* were amplified by polymerase chain reaction (PCR) and subjected to bidirectional sequencing and analysis.

**Results:**

All study patients were Chinese with a mean age of 66.2±9.1 years (range 46–86). There were 77 females (71.3%). Forty-nine out of the one hundred and eight subjects had previous symptomatic PACG, and 59 had asymptomatic PACG. The mean axial length was 21.90±0.50 mm (range 19.98–22.50 mm). We identified a possible disease-causing variant in *CHX10* (c.728G>A) resulting in Gly243Asp substitution in one patient. This variant was not found in 215 normal controls. Several *CHX10* and *MFRP* polymorphisms were also identified.

**Conclusions:**

Our results do not support a significant role for *CHX10* or *MFRP* mutations in PACG.

## Introduction

Glaucoma, a group of heterogeneous optic neuropathies characterized by progressive visual field loss, is the leading cause of irreversible blindness worldwide [1-3]. Categorized according to the anatomy of the anterior chamber angle, there are two main forms of glaucoma, primary open-angle glaucoma (POAG) and primary angle closure glaucoma (PACG). Primary angle closure glaucoma is a major form of glaucoma in Asia, especially in populations of Chinese and Mongoloid descent [4-9] compared to primary open-angle glaucoma, which is the predominant glaucoma disease among Caucasians and Africans [10,11]. PACG is responsible for substantial blindness in Mongolia [6], Singapore [7], China [8,9], and India [12,13]. It is estimated that PACG blinds more people than POAG worldwide [8].

Glaucoma has a major genetic basis, estimated to account for at least a third of all glaucoma cases [14-16]. Although several genes have been identified for POAG [17-19], the gene(s) underlying PACG is still unknown. Eyes with PACG tend to share certain anatomic biometric characteristics. These include a short axial length of the eyeball, shallow anterior chamber depth, hyperopia, and a thicker and more anteriorly positioned lens compared to the rest of the population [20-24]. The association with smaller ocular dimensions makes ocular developmental genes possible candidate genes for the condition. Eyes with microphthalmia and nanophthalmos are characterized by very short axial length, high hypermetropia, high lens/eye volume ratio, and a high prevalence of angle closure. Intraocular pressure is greatly elevated in many cases. Recently, two “small eye” genes have been identified. Non-syndromic microphthalmia was associated with mutations in the retinal homeobox gene *CHX10* [25,26]. Sundin et al. [27] found that null mutations in *MFRP* , which encodes a Frizzled related protein that regulates axial length, results in extreme hyperopia, and nanophthalmos.

To investigate the possible involvement of *CHX10* and *MFRP* in PACG, we sequenced both genes in a sample of PACG patients with small ocular dimensions.

## Methods

### Patients

Subjects with PACG were recruited from the glaucoma service of the Singapore National Eye Centre and National University Hospital (Singapore). Written informed consent was obtained from all subjects, and the study had the approval of the ethics committees of the two hospitals and was performed according to the tenets of the Declaration of Helsinki. Standardized inclusion criteria for PACG were used, which were as follows:

1. The presence of glaucomatous optic neuropathy, which was defined as disc excavation with loss of neuroretinal rim tissue with a cup:disc ratio of 0.7 or greater when examined with a 78D biomicroscopic lens.

2. Visual field loss detected with static automated white-on-white threshold perimetry (program 24–2 SITA, model 750, Humphrey Instruments, Dublin, CA) that was consistent with glaucomatous optic nerve damage. This was defined as Glaucoma Hemifield test outside normal limits and/or an abnormal pattern standard deviation with p<0.05 occurring in the normal population.

3. A closed angle on indentation gonioscopy. A closed angle was defined as an angle of at least 180 degrees in which the posterior pigmented trabecular meshwork was not visible on gonioscopy.

4. We only included eyes with axial lengths less than 22.5 mm. Axial length measurements were performed by A-mode applanation ultrasonography (Sonomed A2500, Haag-Streit, Koniz, Switzerland).

Subjects were further categorized into two groups, those who presented with acute symptomatic angle-closure and those who had asymptomatic PACG. Characteristics of the acute angle closure episode were obtained from the charts retrospectively. For this study, acute angle-closure was defined as follows:

1. Presence of at least two of the following symptoms: ocular or periocular pain, nausea, and/or vomiting, an antecedent history of intermittent blurring of vision with haloes.

2. An intraocular pressure (IOP) of more than 28 mmHg (as measured by Goldmann applanation tonometry) and the presence of at least three of the following signs: conjunctival injection, corneal epithelial edema, mid-dilated unreactive pupil, shallow anterior chamber, glaucomflecken, and iris atrophy.

Ninety-three normal controls were screened in this study. Normal control subjects had open angles, intraocular pressures of less than 21 mmHg, axial lengths greater than 23 mm, normal optic nerve heads with cup:disc ratio of less than or equal to 0.5, normal Heidelberg Retinal Tomography scans, and no other ocular pathology or family history of glaucoma.

### Mutational analysis

Genomic DNA was extracted from leukocytes of the peripheral blood samples, and exons of *CHX10* and *MFRP* were amplified by polymerase chain reaction (PCR) with the DNA Theromocycler 9700 (Applied Biosystems, Foster City, CA). PCR reactions were performed in 50 µl reaction volumes containing 10 mM TrisHCl (pH 8.9), 50 mM KCl, 1.5 mM MgCl_2_, 25 pmoles of each primer, 200 μM of each dNTP, 50–100 ng of patient genomic DNA, and 0.7 units of Taq thermostable DNA polymerase (Promega, Madison, WI). Cycling parameters were 3 min at 95 °C followed by 35 cycles of 30 s at 95 °C, 30 s at the melting temperature (T_m_) of the primers (52-62 °C), and 30 s at 72 °C with a final 5 min extension at 72 °C. PCR products were purified using GFX PCR clean up columns (Amersham Biosciences, Piscataway, NJ). Sequence variations were identified by automated bidirectional sequencing using BigDye terminator v3.1 chemistries (Applied Biosystems). An automated DNA sequencer (Model, ABI PRISM 3100, Applied Biosystems) was used. Primers for sequence reactions were the same as those for the PCR reaction.

Statistical analysis was performed using the χ^2^/Fisher’s Exact tests (SPSS 11.5).

## Results

A total of 108 subjects with PACG were studied of which 49 subjects presented with acute angle closure while 59 eyes had asymptomatic PACG. All subjects were of Chinese ethnicity. There were 77 females (71%), and the mean age was 66.2±9.1 years (range 46–86 years; Table 1).

**Table 1:** Demographic characteristics and ocular dimensions of primary angle closure glaucoma subjects in this study.

**Characteristic**	**Overall (n=108)**	**Previous symptomatic PACG (n=49)**	**Asymptomatic chronic PACG (n=59)**
**Sex**			
Male	31 (28.7%)	10	21
Female	77 (71.3%)	39	38
**Age (years)**			
Mean±SD	66.2±9.1	64.2±8.8	67.8±9.0
range	(46–86)		
**Axial length (mm)**			
Mean±SD	21.90±0.50	21.79±0.58	21.99±0.40
(range)	(19.98–22.50)		
**Anterior chamber depth (mm)**			
Mean±SD	2.37±0.38	2.44±0.45	2.32±0.31
(range)	(1.79–3.54)		

The demographic features and ocular dimensions of the 108 Chinese PACG study subjects are shown. Only patients with axial lengths less than 22.5mm were included in the study. SD: Standard deviation.

**Figure 1:**
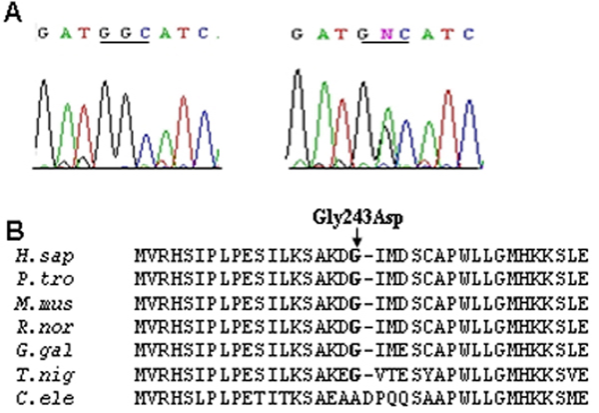
The c.728G>A (Gly243Asp) variation in *CHX10*. **A**: The wild type sequence is shown on the left to compare with the variant *CHX10* sequence on the right. The comparison depicts the G>A transition that changed codon 243 from glycine (GGC) in the wild type sequence to aspartic acid (GAC) in the variant* CHX10* sequence. **B**: Protein alignment of human (*Homo sapiens*) CHX10 (residues 215-261) is compared to other CHX10-like proteins from other species, i.e., chimp (*P. troglodytes*), mouse (*M. musculus*), rat (*R.norvegicus*), chick (*G. gallus*), puffer fish (*T. nigroviridis*), and worm (*C. elegans*). This comparison shows the conservation of the glycine 243 residue in *CHX10*.

Four *CHX10* sequence alterations consisting of two heterozygous missense and two synonymous changes were identified in our study subjects (Table 2). The two synonymous variations, Ser157Ser and Pro250Pro, were both found in control subjects. The missense sequence alteration, Asp291Asn (D291N), identified in seven PACG cases, was also detected in eight normal controls. The Gly243Asp (728G>A) missense change was found in one patient with previous acute PACG (who also had the Arg257His mutation in *MFRP*, see below) but not in a total of 215 controls of Chinese ethnicity (Figure 1A). We analyzed an additional 122 control subjects for this particular sequence variant apart from the initial 93 control subjects enrolled in the study. The Gly243Asp (728G>A) missense change also involved a residue that is conserved across several species (Figure 1B). The patient concerned was an 82-year-old lady with chronic PACG first diagnosed at age 75. At presentation, the vertical cup:disc ratio was 0.8 in both eyes, and gonioscopy revealed 360 degrees of closed angles with extensive peripheral anterior synechiae. The axial length was 21.05 mm, and anterior chamber depth was 2.09 mm in the affected eye.

**Table 2:** *CHX10* and *MFRP* sequence alterations detected and investigated in primary angle closure glaucoma patients.

**Gene**	**Sequence change**	**Exon**	**Allele Distribution (%)**			**Allele Association (p-value)**	**Odds ratio (95% CI)**	**Genotype Distribution (%)**			**Genotype Association on p-value**	**Odds ratio (95% CI)**
				**Cases n=108**	**Controls n=93**				**Cases n=108**	**Controls n=93**		
*CHX10*	Ser157Ser (c.471C>T) rs35435463	Exon 3	C	206 (0.95)	177 (0.95)	1.0000	1.05 (0.37–2.94)	CC	99 (0.92)	84 (0.903)	0.6187	1.32+ (0.43–4.14)
			T	10 (0.05)	9 (0.05)			CT	8 (0.074)	9 (0.097)	NA	NA
								TT	1 (0.009)	0 (0.0)	NA	NA
	*Gly243Asp (c.728G>A)	Exon 4	G	215 (0.995)	186 (1.00)	NA	NA	GG	107 (0.99)	93 (1.0)	NA	NA
			A	1 (0.005)	0 (0.0)			GA	1 (0.01)	0 (0.0)	NA	NA
								AA	0 (0.0)	0 (0.0)	NA	NA
	Pro250Pro (c.750G>A)	Exon 4	G	215 (0.995)	180 (0.97)	0.0525	7.14 (0.85–330.62)	GG	107 (0.99)	87 (0.94)	0.0506	7.32+ (0.86–341.96)
			A	1 (0.005)	6 (0.03)			GA	1 (0.01)	6 (0.06)	NA	NA
								AA	0 (0.0)	0 (0.0)	NA	NA
	Asp291Asn (c.871G>A)	Exon 5	G	209 (0.97)	178 (0.96)	0.6073	1.34 (0.42–4.44)	GG	101 (0.94)	85 (0.91)	0.6005	1.35+ (0.41–4.59)
			A	7 (0.03)	8 (0.04)			GA	7 (0.06)	8 (0.09)	NA	NA
								AA	0 (0.0)	0 (0.0)	NA	NA
*MFRP*	Arg64Arg (c.192C>G)	Exon 3	C	196 (0.99)	177 (0.99)	1.0000d	0.55 (0.01–10.74)	CC	97 (0.98)	88 (0.99)	1.0000d	0.55+ (0.01–10.79)
			G	2 (0.01)	1 (0.01)			CG	2 (0.02)	1 (0.01)	NA	NA
								GG	0 (0.0)	0 (0.0)	NA	NA
	Val136Met (c.406G>A) rs3814762	Exon4	G	185 (0.86)	157 (0.84)	0.5725	1.18 (0.65 - 2.14)	GG	79 (0.74)	65 (0.70)	0.6316	1.21+ (0.62 - 2.38)
			A	29 (0.14)	29 (0.16)			GA	27 (0.25)	27 (0.29)	1.0000D	1.21++ (0.01 - 96.52)
								AA	1 (0.01)	1 (0.01)	1.0000d	1.00+++ (0.01 - 81.32)
	Tyr164Tyr (c.492C>T) rs36015759	Exon 5	C	164 (0.77)	148 (0.80)	0.5455	0.84 (0.51–1.39)	CC	64 (0.60)	58 (0.62)	1.0000d	0.98+ (0.52 - 1.85)
			T	50 (0.23)	38 (0.20)			CT	36 (0.34)	32 (0.35)	0.3394	0.47++ (0.07 - 2.20)
								TT	7 (0.06)	3 (0.03)	0.498	0.49+++ (0.07- 2.36)
	His180His (c.540 C>T) rs2510143	Exon 5	C	166 (0.82)	163 (0.88)	0.1575	0.65 (0.35 - 1.18)	CC	69 (0.68)	72 (0.78)	0.2397	0.65+ (0.31 - 1.33)
			T	36 (0.18)	23 (0.12)			CT	28 (0.28)	19 (0.20)	0.4417	0.48++ (0.04 - 3.48)
								TT	4 (0.04)	2 (0.02)	1.0000d	0.74+++ (0.06–5.78)
	*Arg257His (c.770 G>A)	Exon 6	G	208 (0.99)	186 (1.0)	NA	NA	GG	103 (0.98)	93 (1.0)	NA	NA
			A	2 (0.01)	0 (0.0)			GA	2 (0.02)	0 (0.0)	NA	NA
								AA	0 (0.0)	0 (0.0)	NA	NA
	Leu318Leu (c.954 G>A) rs35885438	Exon 9	G	186 (0.98)	178 (0.96)	0.2547	2.08 (0.55–9.63)	GG	91 (0.96)	85 (0.91)	0.2473	2.13+ (0.55–10.04)
			A	4 (0.02)	8 (0.04)			GA	4 (0.04)	8 (0.09)	NA	NA
								AA	0 (0.0)	0 (0.0)	NA	NA

Allele and genotype frequencies of sequence variations within *CHX10* and *MFRP *genes in Chinese PACG patients versus Chinese control subjects. The observed genotype distributions did not deviate from those predicted by the Hardy–Weinberg equilibrium. Proportions of groups were compared by the Fisher’s exact test. The criterion for statistical significance was p≤0.05.  DNA changes are documented based on cDNA sequences with +1 corresponding to the A of the ATG translation initiation codon in reference sequences NM_182894.1 (*CHX10*) and NM_031433.1 (*MFRP*). The asterisks represent Gly243Asp in *CHX10* and Arg257His in *MFRP* that are rare putative pathogenic variants. Exon 4 of *CHX10* was screened in an additional 122 control subjects to investigate the Gly243Asp variant. The vertical arrow (+) represents odds ratios when comparing likelihood of PACG in individuals with two copies of major allele versus one copy. Double plus (++) represents odds ratios when comparing likelihood of PACG in individuals with two copies of the major allele versus individuals with no copies of the major allele. Triple plus (+++) represents odds ratios when comparing likelihood of PACG in individuals with one copy of major allele versus no copies of the major allele.

**Figure 2:**
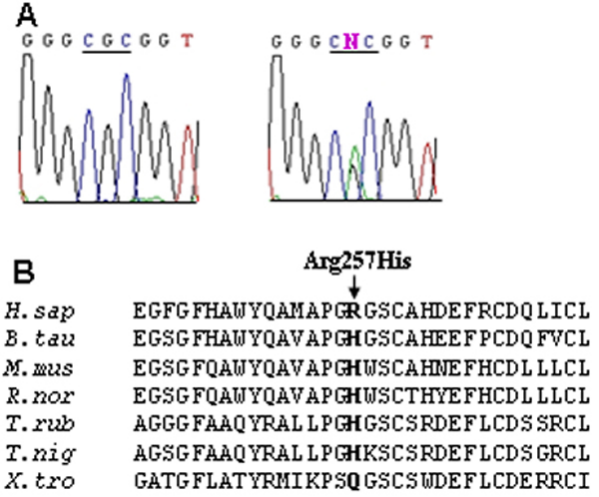
The c.770G>A (Arg257His) variation in *MFRP*. **A**: The wild type sequence is shown on the left to compare with the variant *MFRP* sequence on the right. The comparison depicts the G>A transition that changed codon 257 from arginine (CGC) in the wild type sequence to histidine (CAC) in the variant *MFRP* sequence. **B**: Protein alignment of human (*H. sapiens*) MFRP (residues 242-273) is compared to other MFRP-like Proteins from other species, i.e., cow (*B. taurus*), mouse (*M. musculus*), rat (*R. norvegicus*), fugu (*T. rubripes*), puffer fish (*T. nigroviridis*), and *Xenopus* (*X. tropicalis*).  This comparison shows the conservation of the arginine 257 residue of *MFRP*.

Two missense changes (Val136Met and Arg257His) were identified in *MFRP*. The Arg257His variation (Figure 2A) that was identified in only two PACG cases was not detected in 93 controls. In homologous sequences, histidine residue is also seen in place of the arginine residue (Figure 2B). One of the patients with the Arg257His change in *MFRP* also had the rare Gly243Asp variation in *CHX10.* Four common silent *MFRP* variations were also identified (Arg64Arg, Tyr164Tyr, His180His, and Leu318Leu). The distribution of these variants between PACG and controls was not statistically significant.

## Discussion

There are few studies to date on the genetics of PACG, although transmission via a single, dominant gene has been suggested [28]. However, there are various published studies on PACG that suggest a genetic basis for the condition. First, ocular characteristics related to angle closure glaucoma are more common in close relatives of affected patients than in the general population; these characteristics include short axial lengths of the eyeball and shallow anterior chambers [29-33]. First-degree relatives of subjects with PACG have a three to five times greater risk of developing the disorder compared with the general population [29,30,32,34]. There are also ethnic differences with a higher prevalence among Inuits (2%-8%) and Asians (0.3%-1.4 %) compared to Caucasians (0.1%), suggesting a genetic predisposition to the disorder [35]. Although these studies have suggested a familial risk of angle closure, the precise heritability of the condition is largely unknown. The familial risk of PACG has also not been determined in Asian populations in which the prevalence of the disease is high.

Due to the association of PACG with small ocular dimensions, we investigated the role of two genes associated with small eyes, *CHX10* and *MFRP*, in a cohort of 108 PACG subjects all of whom had axial lengths less than 22.5 mm. This cut-off was arbitrarily chosen based on the results of a population-based study in Singapore that found that the mean axial length of adults aged 40 years or older was 23.23±1.17 mm [36]. Recently, another Singapore study found that the mean axial length of subjects with angle closure was 23.02 mm compared to 23.85 mm in normal subjects [37]. However, the results of this study indicate that *CHX10* and *MFRP* mutations do not play a major role in the causation of chronic PACG as most of the sequence variations found are not thought to be disease-causing. Nevertheless, to firmly exclude a role of these genes in PACG, a larger case-control study may be required. We found several sequence variants in *CHX10* including two silent polymorphisms (Ser157Ser and Pro250Pro) and the missense change, Asp291Asn, all of which were found in control subjects. In *MFRP*, we identified four silent variations (Arg64Arg, Tyr164Tyr, His180His, and Leu318Leu) and a missense variation (Val136Met), which all appear to be common *MFRP* polymorphisms. Interestingly, one PACG subject had both the Gly243Asp missense change in *CHX10* and the Arg257His variation in *MFRP.* However, Arg257His is a conservative substitution and is unlikely to be pathogenic given that in homologous sequences the histidine residue is also seen in place of the arginine residue (Figure 2B). The conservation of the *CHX10* Gly243 residue in homologous sequences (Figure 1B) and the absence of Gly243Asp in more that 400 normal chromosomes favor it being a disease-causing variant rather than a polymorphism. However, we have not been able to demonstrate segregation of Gly243Asp with the disease in the proband’s family, mostly due to the late onset nature of the disease. To our knowledge, there is no evidence of family history of disease in the proband’s family. Therefore, in the absence of any other convincing mutations in *CHX10* for PACG, one cannot discount the possibility of Gly243Asp still being a rare nonpathogenic variant.

PACG can also be a slowly progressive disease process that can be accelerated by an enlarging cataractous lens from middle age onwards. The axial lengths of patients with PACG appear to be shorter than the normal population but not as short as nanophthalmic eyes. It can be speculated that PACG eyes may form one end of the large spectrum of structural anomalies seen in both microphthalmos and nanophthalmos, making both *CHX10* and *MFRP* good candidate genes for PACG. However, our results did not support a significant role for *CHX10* or *MFRP* mutations in PACG.

There has been a paucity of research into the genetic basis of PACG. To date, a genetic locus for PACG has not been published, and there have been few other reports of candidate gene association studies related to PACG. This is probably related to the high prevalence of the condition in populations where glaucoma research has not been a major focus. There may be under-reporting of a family history as in POAG that may lead to the impression that most affected patients are isolated cases. The late onset of the disease and the lack of accurate clinical information on previous generations are further obstacles to determining the genetics of the disorder. It is hoped that further research into the genetic basis of PACG would lead to improved understanding of the molecular basis of this major cause of world blindness.
